# The relationship between physical activity and anxiety in college students: exploring the mediating role of lifestyle habits and dietary nutrition

**DOI:** 10.3389/fpsyg.2024.1296154

**Published:** 2024-06-21

**Authors:** Dezhuo Sun, Xiangfei Zhu, Zhonghan Bao

**Affiliations:** ^1^Faculty of Education, Silpakorn University, Nakhon Pathom, Thailand; ^2^Faculty of Physical Education, Putian University, Putian, China

**Keywords:** college students, physical activity, lifestyle habits, dietary nutrition, anxiety

## Abstract

**Background:**

Physical activity has been shown to be effective in treating and improving anxiety in college students. However, no studies have been conducted to examine the relationship between physical activity and anxiety in college students through mediating factors such as dietary nutrition and lifestyle habits. Therefore, the aim of this study was to examine the mediating role of lifestyle and dietary nutrition in the relationship between physical activity and anxiety.

**Methods:**

This study used a stratified random sampling method to survey 498 college students from three universities in Fujian, China. Data on participants’ demographic characteristics, physical activity, lifestyle habits, and dietary nutrition were collected and analyzed using SPSS software. The proposed structural equation model was analyzed using Amos software.

**Results:**

The results of the study showed that dietary nutrition and lifestyle habits had significant independent mediating effects and continuous multiple mediating effects (*p* < 0.01) in the effects of physical activity on college students’ anxiety. Dietary nutrition and lifestyle habits played an independent mediating role, accounting for 24.9% of the total effect; there was also a continuous multiple mediating effect between dietary nutrition and lifestyle habits, accounting for 13.27% of the total effect value. In addition, physical activity had a direct effect value on anxiety in college students, accounting for 36.93% of the total effect value.

**Conclusion:**

By increasing the behavior and awareness of college students to participate in physical activity, supplemented by guiding them to develop regular lifestyle habits and correct dietary nutritional patterns, the anxiety level of college students can be effectively improved and reduced. This type of regulation is an important reference for the self-management and rehabilitation of college students with anxiety disorders. Future studies can experimentally develop a combined intervention of physical activity, lifestyle habits, and dietary nutritional to help college students better cope with anxiety.

## 1 Introduction

Anxiety disorders are one of the most common mental health disorders today (Aucoin et al., 2021) and one of the most common mental health problems among Chinese college students, with a detection rate of approximately 31% (Chang et al., 2021). Studies have reported that even when anxiety levels are below psychiatric diagnostic thresholds, college students are vulnerable to risks such as substance abuse and obesity, and severe anxiety situations can lead to suicidal behavior (Wolitzky-Taylor et al., 2012; Bentley et al., 2016). Furthermore, according to a nationally representative US survey, people with anxiety disorders have a life expectancy that is 7.9 years shorter than people without anxiety disorders (Pratt et al., 2016). Given the seriousness of anxiety disorders, scientists have conducted in-depth research on anxiety disorders in college students with the goal of better identifying and intervening to help alleviate and reduce anxiety levels in college students.

### 1.1 Physical activity and anxiety

Recent studies suggest that physical activity may be beneficial in the treatment of anxiety disorders (Carter et al., 2021; Marconcin et al., 2022; Li et al., 2023). Existing evidence strongly suggests that physical activity interventions can broadly reduce anxiety symptoms in the population (Kandola et al., 2018; Ji et al., 2022). A randomized controlled trial showed a significant reduction in anxiety symptoms in the experimental group that participated in the exercise trial with a significant effect (Stubbs et al., 2017). Active people are less likely to suffer from anxiety disorders (Liu et al., 2023), and in a survey of 7,874 people it was found that moderate and high levels of physical activity significantly reduced anxiety symptoms compared to light physical activity (Mc Dowell et al., 2020).

Conversely, low physical activity and high screen time increase the risk of mental health problems among college students (Wu et al., 2015). College students experience significant increases in stress, anxiety, and depression due to prolonged exposure to sedentary behaviors such as studying, computer games, smartphones, and television (Buckworth and Nigg, 2004; Lee and Kim, 2019). Previous studies have shown that physical activity is low cost, high compliance, low side effects, and has significant benefits (Hallgren et al., 2017; Kandola et al., 2018) to prevent and improve anxiety (Carter et al., 2021). Based on the above findings, hypothesis 1: physical activity positively affects the reduction and improvement of anxiety symptoms in college students.

### 1.2 The mediating role of dietary nutrition

Dietary nutrition is the foundation for good health and functioning and is essential for human growth and development, as well as playing an important role in the prevention and treatment of mental health disorders (Ohlhorst et al., 2013). A healthy food-based dietary model may help prevent and treat health disorders such as anxiety (Kris-Etherton et al., 2021). A controlled trial of dietitian-led improvements in the dietary quality of patients with anxiety disorders showed that dietary nutrition significantly improved patients’ symptoms (Forsyth et al., 2015). However, some students with less self-control may overeat, increasing the risk of overweight or obesity (Anton-Păduraru et al., 2021). In addition, dietary nutrition has been used to regulate dietary quality to play an important moderating role between sleep and mental health (Zhao et al., 2021).

It has been shown that physical activity has been identified as a method that can lead to healthier eating behaviors and the regulation of eating behaviors (Fernandes et al., 2023). In one triai, 15 weeks of exercise training was found to motivate young people to pursue a healthier diet and regulate their food intake (Joo et al., 2019). An Austrian survey of 52 universities across the country found that students who participated in physical activity had healthier eating behaviors than those who did not (Wirnitzer et al., 2023). Physical activity is effective in controlling and preventing a number of diseases caused by overweight and obesity due to poor nutrition (Morgan et al., 2020). Therefore, combined interventions of diet and nutrition and physical activity may be more effective in improving mental disorders in students (Glozah et al., 2018). In fact, in a randomized controlled triai., researchers found that an intervention group of adaptive physical activity combined with diet had a significant effect on reducing anxiety symptoms at week 18 (Forsyth et al., 2015). This suggests that better nutritional status and moderately active physical activity are associated with reduced levels of anxiety symptoms (Chi et al., 2021). Therefore, both physical activity and dietary nutrition contribute to the physical health of college students (Hosker et al., 2019). Because previous studies have shown a strong relationship between physical activity and dietary nutrition and between dietary nutrition and anxiety. Therefore, a second hypothesis was proposed: dietary nutrition mediates the relationship between physical activity and anxiety in college students. Therefore, hypothesis 2 is proposed as: dietary nutrition mediates the relationship between physical activity and anxiety in college students.

### 1.3 The mediating role of lifestyle habits

There is a strong link between good lifestyle habits and physical and mental health. Studies have shown that there is a positive correlation between lifestyle habits such as regular eating, adequate sleep, and regular rest and relaxation and anxiety among college students (Ramón-Arbués et al., 2020; Chellappa and Aeschbach, 2022; Xiao et al., 2022). Good habits can help reduce anxiety. Conversely, negative changes in lifestyle habits increase the risk of psychiatric disorders such as anxiety and depression (Blom et al., 2021). Research has also shown that factors such as sleep disturbance and changes in eating habits have a direct impact on mental health status and decreased quality of life (Caroppo et al., 2021). There is no doubt that healthy habits are crucial for mental health (Tada, 2017). Healthy lifestyle habits play a key role in explaining the mediating effects between various predictors and mental health. Some studies have shown that lifestyle habits have a moderating effect on the relationship between spirituality and mental health (Bożek et al., 2020). Recent research has also found that healthy lifestyle habits moderate the relationship between psychosocial dysfunction and mental health (Rico-Bordera et al., 2023).

A large body of research suggests that regular exercise can help regulate circadian rhythms and develop good lifestyle habits that improve sleep health, metabolism, and immune function (Lewis et al., 2018; Youngstedt et al., 2019; Shen et al., 2023). People who are physically active tend to get enough sleep and adopt good eating habits (Kim et al., 2016; Kiebuła et al., 2020; Min et al., 2021). Moreover, physical activity has been shown to counteract or even exceed the detrimental effects of some poor dietary habits, sleep deprivation, and irregular work and rest schedules on physical and mental health (Zheng et al., 2015; Wei et al., 2022). It can be seen that physical exercise for college students can develop life habits such as good eating habits, quality sleep, and regular work and rest. Therefore, hypothesis 3 was the mediating variable of lifestyle habits in the relationship between physical activity and anxiety in college students.

### 1.4 Sequential mediating role of lifestyle habits and dietary nutrition

There is a positive correlation between a healthy lifestyle and mental health and well-being. Research has shown that a healthy lifestyle can be effective in reducing anxiety in college students (Hoying et al., 2020; Hautekiet et al., 2022). In contrast, unhealthy lifestyles are positively associated with symptoms of depression and anxiety, and evidence suggests that sleep deprivation, excessive sleep, lack of physical activity, and poor diet all contribute to the risk of developing anxiety (Bonnet et al., 2005; Zhang et al., 2023; Tang et al., 2024). While physical activity behaviors, dietary nutrition, and lifestyle habits are important components of a healthy lifestyle for college students (Wang et al., 2012; Bao et al., 2022), each plays a unique role in influencing college students’ anxiety levels, but they are also intrinsically linked. It has been suggested that moderate dietary nutrition may promote healthy eating habits, enhance regulation of circadian rhythms, and form good lifestyle habits (Golem et al., 2014; Egg et al., 2020). Based on the above analysis, maintaining healthy lifestyle habits and quality dietary nutrition work together to further reduce anxiety levels. However, further empirical studies are needed to verify whether using lifestyle habits and nutritional diet as successive multiple mediating variables can more effectively promote the beneficial effects of physical activity in the treatment of anxiety disorders. Therefore, hypothesis 4 was the continuous mediating role of dietary nutrition and lifestyle habits in the relationship between physical activity and anxiety in college students is proposed.

**Figure 1:**
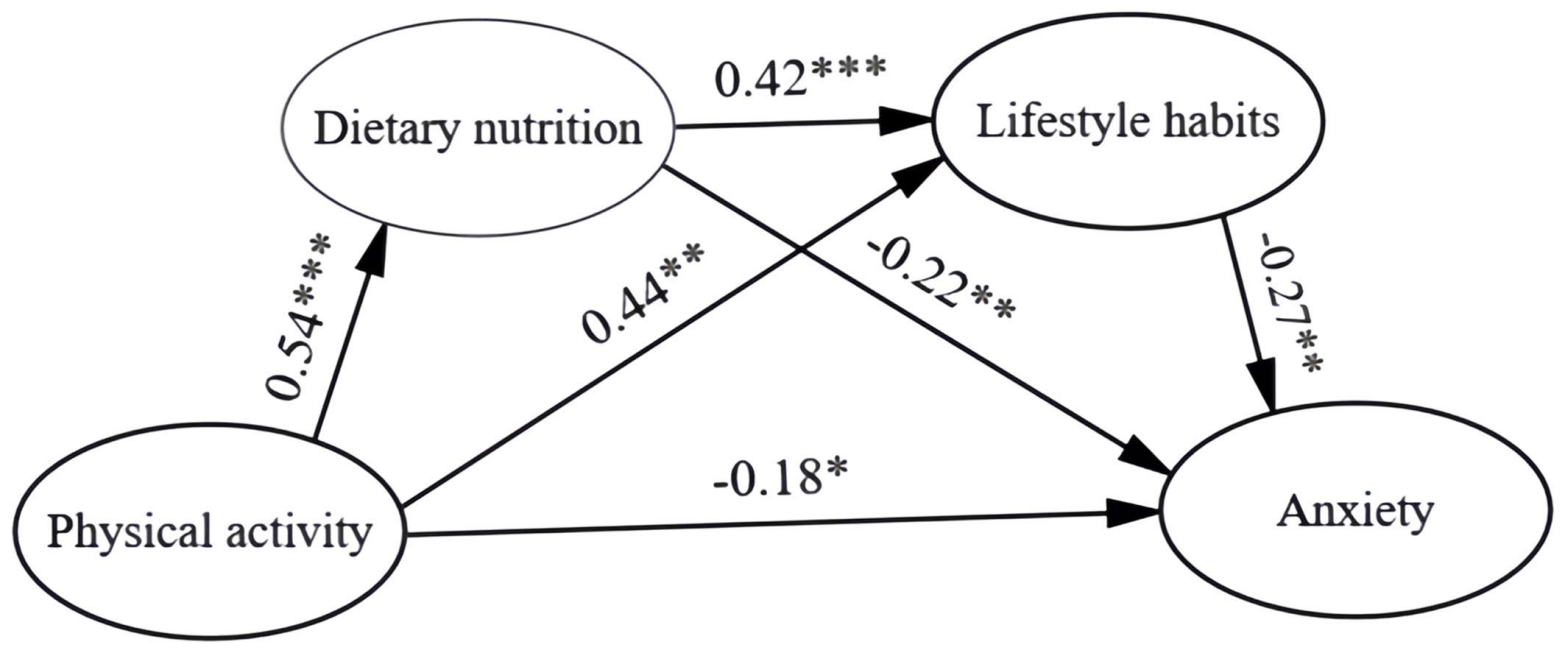
Structural equation modelling of the effects of physical activity on anxiety in college students. ^*^p < 0.05, ^**^p < 0.01, ^***^p < 0.001.

In recent years, the effects of physical activity on anxiety disorders have been widely reported. However, current research has not yet explored the relationship between dietary nutrition and lifestyle habits in the relationship between physical activity and anxiety in college students. Therefore, the present study proposes a mediation model (Figure 1) to further explore the relationship between physical activity, dietary nutrition, lifestyle habits and anxiety among college students, and to provide theoretical guidance for alleviating and improving college students’ anxiety disorders.

## 2 Methods

### 2.1 Study design

In this study, students from three universities in Fujian Province were selected as respondents. To ensure the representativeness of the sample, this study used stratified random sampling. Since juniors and seniors do not have a regular physical education program, they were excluded from the survey. The sample size for each school and grade level was determined by calculating each school’s percentage of freshmen and sophomores at the three universities. Inclusion criteria for the sample included (1) voluntary participation in the survey and informed consent, and (2) being a college student. Exclusion criteria were as follows: (1) the questionnaire was incomplete; (2) there were logical errors in the responses. Based on these criteria, we identified a sample that met the survey requirements.

This study began in June 2023 and 540 students were recruited to participate voluntarily. However, 42 questionnaires were excluded due to incomplete or identical responses, resulting in a valid sample of 498 (92.2% participation rate).

### 2.2 Measures

#### 2.2.1 Physical activity

The physical activity scale used in this study is based on Wang et al.’s (2012) development of the sport and exercise behavior dimension. The scale consists of three items in which participants answer the questions “Can you do more vigorous exercise for 30 min at least three times a week?,” “Do you do warm-up activities before each exercise?,” “Do you do warm-up activities before each exercise?, “Do you warm up before each exercise session?” and “Do you engage in chronic aerobic exercise for 30–60 min three times a week?” on a 5-point Likert scale ranging from 1 (never) to 5 (all), with higher scores indicating higher levels of exercise among college students. The scale has been validated for reliability and validity in previous studies. In this study, the Cronbach’s alpha coefficient for the scale was 0.791, indicating that the physical activity scale has good reliability.

#### 2.2.2 Lifestyle habits

The Lifestyle Habits Scale references the Regular Lifestyle Behavior dimension developed by Wang et al. (2012). Participants answered the following questions: “Do you eat three regular meals a day?,” “Do you have a regular start time each day?,” “Do you get enough sleep each day?.” Participants responded to the question “Do you get enough sleep every day?” on a 5-point Likert scale ranging from 1 (never) to 5 (all the time), with higher scores indicating better living habits among college students. The scale has been validated for reliability and validity in previous studies. In this study, the Cronbach’s alpha coefficient for the Life Habits Scale was 0.743, indicating that the scale has good reliability.

#### 2.2.3 Dietary nutrition

The Dietary Nutrition Scale uses the Dietary Nutrition Behavioral Dimensions developed by Wang et al. (2012). The scale contains four items: “Do you eat breakfast every day? Do you drink at least about 800 mL of water every day? “Do you consciously choose light and less salty foods? “Do you eat foods rich in vitamins (e.g., fruits, vegetables, etc.)?.” The scale is rated on a 5-point Likert scale ranging from 1 (never) to 5 (all), with higher scores indicating a more nutritionally balanced diet among college students. The reliability and validity of the scale have been validated in previous studies. In this study, the Cronbach’s alpha coefficient for the Dietary Nutrition Scale was 0.707, indicating that the scale has good internal consistency.

#### 2.2.4 Anxiety

The Generalized Anxiety Scale (GAD-7) was used in this study to assess symptoms of anxiety in college students (Spitzer et al., 2006). This scale is considered the most accurate measure of generalized anxiety disorder in college students (Swinson, 2006; Manzar et al., 2021). The scale consisted of seven symptoms experienced in the past two weeks, such as “In the past two weeks, you have not felt nervous, anxious, or eager, “to which participants responded on a 4-point Likert scale ranging from 0 (none) to 3 (almost every day). The scale is based on a 4-point Likert scale with a score range of 0–3. A total score of 0 to 4 indicates normai., 5 to 9 indicates mild anxiety, 10 to 14 indicates moderate anxiety, and > 15 indicates severe anxiety. The reliability and validity of this scale have been validated in previous studies. The Cronbach’s alpha coefficient for the scale in this study was 0.910.

### 2.3 Data analysis

All data are analyzed using IBM SPSS version 21 software (IBM Corporation, Armonk, NY, United States) and Amos version 26 software (IBM Corporation, Armonk, NY, USA). In the first step, we will analyze the frequency counts of the participants’ demographics and the reliability of the scales used for each variable. We used T-tests to examine the variability of each variable between students with and without anxiety disorders, and Pearson correlation analyses to test for correlations between the variables. In the second step, this study will use Amos software to construct a structural equation model and confirmatory factor analysis to examine standardized utility metrics for each pathway. The bootstrap method was used to evaluate the mediation effect.

## 3 Results

### 3.1 Demographic characteristics of the participants

Among the invited respondents (as shown in Table 1), there were 236 males (47.4%) and 262 females (52.6%), aged between 18 and 20 years. In terms of self-reported health status, only 92 students (19.5%) reported being very healthy, while 144 students (28.9%) reported suffering from chronic diseases such as rhinitis and gastritis. In addition, 142 students (28.5%) were diagnosed with mild anxiety, while 8 students (1.6%) were diagnosed with moderate anxiety.

**Table 1:** Demographic characteristics of participants.

Characteristics		*n*	%
Gender	Male	236	47.4
	Female	262	52.6
Age	18	108	21.7
	19	163	32.7
	20	227	45.6
Self-assessed health status	Relatively unhealthy	25	5
	Fair	172	34.5
	Healthier	209	42
	Very healthy	92	18.5
Chronic disease	No	284	57
	Yes	214	43
Anxiety disorder	No (0–4)	348	69.80
	Mild(5–9)	142	28.5
	Moderate(10–14)	8	1.6

### 3.2 Differential analysis of physical activity, lifestyle habits, and dietary nutrition of students with and without anxiety disorder

According to Table 2, there were significant differences between students with and without anxiety disorders in terms of physical activity (*t* = 6.832, *p* < 0.001), lifestyle habits (*t* = 8.434, *p* < 0.001) and dietary nutrition (*t* = 8.246, *p* < 0.001). The mean physical activity score for students without anxiety (8.90 ± 2.78) was higher than the mean physical activity score for students with anxiety (7.09 ± 2.55). Similarly, the mean of lifestyle habits (9.93 ± 2.39) and dietary nutrition (13.05 ± 2.82) of students without anxiety disorder were higher than the mean of lifestyle habits (7.91 ± 2.57) and dietary nutrition (10.73 ± 3.02) of students with anxiety disorder.

**Table 2:** Differential analysis of physical activity, lifestyle habits, and dietary nutrition of students with and without anxiety disorder.

	Anxiety (Mean ± SD)		*t*	*p*
	No (*n* = 348)	Yes (*n* = 150)		
Physical activity	8.90 ± 2.78	7.09 ± 2.55	6.832	<0.001^***^
Lifestyle habits	9.93 ± 2.39	7.91 ± 2.57	8.434	<0.001^***^
Dietary nutrition	13.05 ± 2.82	10.73 ± 3.02	8.246	<0.001^***^

^***^p < 0.001.

### 3.3 Correlation analysis between college students’ physical exercise, living habits, diet and nutrition and anxiety

According to the results in Table 3, there is a significant negative correlation (*p* < 0.01) between physical activity, lifestyle habits and dietary nutritional behaviors and anxiety among college students. There was also a significant positive correlation between physical activity, lifestyle habits and dietary nutrition (*p* < 0.01).

**Table 3:** Correlation analysis between college students’ physical exercise, living habits, diet nutrition and anxiety.

Variables	Physical activity	Lifestyle habits	Dietary nutrition	Anxiety
Physical activity	1.000			
Lifestyle habits	0.544^**^	1		
Dietary nutrition	0.437^**^	0.535^**^	1	
Anxiety	−0.415^**^	−0.459^**^	−0.410^**^	1

^**^Significantly correlated at the 0.01 level (bilateral).

### 3.4 Structural modelling

In this study, AMOS software was used to construct a structural equation model with anxiety as the dependent variable and physical activity, lifestyle habits, and dietary nutrition as the independent variables (as shown in Figure 1). According to the principles and criteria of structural equations (Kline, 2005; Kang et al., 2018), the CMIN/DF of the structural equation model in this study was 2.891 < 0.3, a good fit; RMSEA = 0.062 < 0.08, and all other fit indices, GFI = 0.924, TLI = 0.933, CFI = 0.944, IFI = 0.944, which are all greater than 0.9, indicating that the data and the model are in good agreement. The results showed a significant (*p* < 0.05) path of influence on anxiety levels mediated by a single dietary habit and dietary nutrition. In addition, the path through continuous-multiple mediation between both lifestyle habits and dietary nutrition was also significant (*p* < 0.05), which validated all the hypotheses of this study.

Multiple mediation results of lifestyle habits and dietary nutrition in the relationship between physical activity and anxiety were tested using the Bootstarp method (Table 4). The study showed statistically significant overall indirect benefits of physical activity on anxiety through lifestyle habits and dietary nutrition [estimate = −0.152; 95% CI (−0.221, −0.099), *p* < 0.001]. When mediating variables were considered separately, single mediator mediation for dietary nutrition [estimate = −0.06, 95% CI (−0.116, −0.02)] and single mediator mediation for lifestyle habits [estimate = −0.06, 95% CI (−0.116, −0.019)], as well as continuous-multiple mediator mediation for both dietary nutrition and lifestyle habits [estimate = −0.032, 95% CI (−0.066, −0.011)], were found to be statistically significant (*p* < 0.01).

**Table 4:** Standardization effect and direct effect in the model.

Parameter	Standardized Estimate	Bootstrap 95%CI		***p***	Ratio of effect
		Lower	Upper		
Physical activity→Dietary nutrition→Lifestyle habits→Anxiety	−0.032	−0.066	−0.011	0.002^**^	13.27%
Physical activity→Dietary nutrition→Anxiety	−0.06	−0.116	−0.02	0.003^**^	24.90%
Physical activity→Lifestyle habits→Anxiety	−0.06	−0.116	−0.019	0.006^**^	24.90%
Total Indirect effect	−0.152	−0.221	−0.099	<0.001^***^	63.07%
Direct effect	−0.089	−0.161	−0.02	0.015^*^	36.93%
Total effect	−0.241	−0.304	−0.188	<0.001^***^	

^*^p < 0.05, ^**^p < 0.01, ^***^p < 0.001.

## 4 Discussion

The results of this study have important implications for understanding the relationship between physical activity, lifestyle habits and dietary nutrition and anxiety in college students. From a technical point of view, the study used mediation analysis to reveal the mechanisms of the role of dietary nutrition and lifestyle habits in the relationship between physical activity and anxiety. This approach allowed the researchers to quantify the mediating effects of dietary nutrition and lifestyle habits in the relationship between physical activity and anxiety, and to explain the relationship more precisely. The study also referred to previous research that further supported the mediating role of dietary nutrition and lifestyle habits in the relationship between physical activity and anxiety. This forward-looking approach to research contributes to the coherence and reliability of scientific knowledge. In addition, the innovative and technical nature of this study helps to promote further research and practice in related areas and fills a knowledge gap in the relationship between physical activity and anxiety in terms of lifestyle habits and dietary nutrition.

The results of this study showed that physical activity had a direct positive effect on anxiety, accounting for 36.93% of the total effect. Meanwhile, lifestyle habits and dietary nutrition played independent and continuous multiple mediating roles between physical activity and anxiety, with a total indirect effect of 63.7% of the total effect. These findings provide a scientific basis for interventions for anxiety problems in college students.

It was found that physical exercise has a positive effect on reducing college students’ anxiety, with a direct effect value of −0.089.The direct effect value was −0.089, accounting for 36.93% of the total effect, which is consistent with the results of previous studies (Kayani et al., 2021; Du et al., 2022; Lin and Gao, 2023; Liu and Shi, 2023). Replacing 30 min of sedentary time with 30 min of physical activity has been shown to significantly reduce levels of anxiety symptoms (Dillon et al., 2018). Studies have also found that higher levels of physical activity are associated with significantly lower anxiety symptoms (McDowell et al., 2019; Ji et al., 2022). Physical exercise is effective in increasing cerebral blood flow, increasing the level of neurotrophic factors, improving the function of the hypothalamic–pituitary–adrenal axis, inhibiting the secretion of pro-inflammatory cytokines, and effectively stimulating the central nervous system, thereby alleviating anxiety and other negative emotions (Kruk et al., 2020; Liu et al., 2023). Therefore, we should advocate the relevant departments and educational efforts to fully implement and safeguard the physical activity strategy for college students, and strengthen the publicity and education on physical activity, so as to alleviate and improve the anxiety of college students, and promote the physical and mental health of students.

The study showed that dietary nutrition played an independent mediating role between physical activity and anxiety, accounting for 24.9% of the total effect value. Previous studies have found that physical activity and dietary behaviors have potentially positive effects on psychological distress (Glozah et al., 2018; Ramón-Arbués et al., 2023). It has always been known that physical activity has a reasonable effect on anxiety. However, there is also strong evidence from the nutritional field that symptoms in people with anxiety disorders can be improved by good dietary patterns and diet quality (Faghih et al., 2020; Solomou et al., 2023). A healthy diet provides the body with the nutrients it needs to maintain healthy and stable physiological functions. These nutrients include B vitamins, vitamin C, magnesium and zinc, which play an important role in the synthesis and regulation of neurotransmitters and neurotrophic factors. For example, they promote the synthesis and regulation of neurotransmitters such as serotonin, dopamine, and norepinephrine, which in turn regulate mood and reduce anxiety symptoms (Młyniec et al., 2017; Kris-Etherton et al., 2021; Simkin et al., 2023). In addition, consumption of beneficial microorganisms and prebiotic fibers may also be beneficial in the treatment of anxiety (Aucoin et al., 2021).

It is worth noting that anxiety symptoms may also affect the quality of college students’ diets (Kundu et al., 2022). By mediating this bidirectional relationship, the intervention effectiveness of a healthy diet alone to intervene in anxiety is limited (Aucoin et al., 2021). There is experimental evidence that interventions combining adaptive physical activity and diet have significant effects on reducing anxiety symptoms (Forsyth et al., 2015). Therefore, improving the nutritional pattern and quality of college students’ diets based on physical activity is a model that is more likely to alleviate and minimize the symptoms of anxiety in college students and avoid the serious health threats posed by anxiety.

More interestingly, the independent mediating role of lifestyle habits between physical activity and anxiety was the same as that of diet and nutrition, accounting for 24.9% of the total effect value The effect of lifestyle habits on anxiety is realized primarily through their impact on the overall health and psychological state of the individual. A regular schedule and adequate sleep can help maintain the body’s biological clock and rhythms, increase the body’s resistance and coping ability, and reduce stress and anxiety (Gupta et al., 2022). In previous studies, there are no studies on the correlation between lifestyle habits in physical activity and anxiety in college students, but there is evidence that physical activity can counteract or even exceed the harmful effects of some bad lifestyle habits, etc. on physical and mental health (Zheng et al., 2015; Wei et al., 2022). According to the superimposed correspondence, when physical exercise is combined with good lifestyle habits, it will certainly be more effective in alleviating or reducing anxiety in college students, which is the same as the results of this study. Research shows that when college students engage in regular physical activity, it can improve anxiety, maintain good habits, and more effectively alleviate or reduce college student anxiety.

Another important finding was that lifestyle habits and dietary nutrition played sequential multiple mediating roles between physical activity and anxiety. Although the benefits of this type of intermediation are relatively small, accounting for only 13.27% of the total effect value, its role cannot be ignored. Previous research has shown that a healthy lifestyle can be effective in reducing anxiety in college students (Hoying et al., 2020; Hautekiet et al., 2022). Physical activity, dietary nutrition, and lifestyle habits are important components of a healthy lifestyle for college students (Wang et al., 2012; Bao et al., 2022). Although each of them works independently, there is bound to be some intrinsic mechanism among the three that jointly affects college students’ anxiety. The results of the present study support this intrinsic mechanism, with physical activity playing the most important role, and dietary nutrition and lifestyle habits playing a mediating role, providing equal effect between the two. Dietary nutrition alone focus more on nutrient intake, while lifestyle habits focus more on daily routines, but together these factors play a role in influencing college students’ anxiety. Therefore, we recommend that college students engage in regular physical activity to increase their body’s metabolism. At the same time, healthy lifestyles and behaviors are developed by maintaining proper eating patterns and quality, as well as good habits, which can improve and reduce feelings of anxiety. These findings provide college students with an effective way to manage anxiety and improve mental health.

When college students face mental health issues such as anxiety, most choose to self-manage and self-heal rather than directly seek professional counseling or medication, which may be related to the high cost of treatment and low levels of anxiety (Blanco et al., 2008; Fortney et al., 2016). This makes the conclusions of this study all the more important. Studies have shown that college students need to be physically active, maintain proper dietary patterns and quality, and maintain good habits, which can improve and reduce student anxiety to some extent. This type of conditioning is not only affordable, but also an important reference for the self-management and rehabilitation of college students with anxiety disorders.

This study also has some limitations. First, the sample was limited to freshmen and sophomores, so the results have limited generalizability and are not representative of all student populations. Second, the questionnaire used to collect the data had fewer questions, and while a simplified questionnaire would have helped improve response rates and sample size, a more multidimensional scale assessing physical activity, lifestyle habits, and dietary nutrition among college students might have allowed for a more comprehensive assessment of the relationship between them and anxiety. Future studies should consider increasing the sample size and using a variety of data collection methods and assessment tools to improve the reliability and external validity of the study. Factors such as grade level, gender, and cultural background should also be considered more fully considered to better understand the effects of physical activity, lifestyle habits, and dietary nutrition on anxiety in college students. Experimental studies could also be added to further validate the relationship between lifestyle habits and dietary nutrition in the relationship between physical activity and anxiety in college students. Overall, despite limitations, this study provides valuable information for our understanding of anxiety in college students and provides a basis for further research and intervention development.

## 5 Conclusion

This study found that students without anxiety disorders significantly outperformed students with anxiety disorders in terms of physical activity, lifestyle habits, and diet nutrition. It was also found that dietary nutrition and lifestyle habits play a single mediating role and a continuous-multiple mediating role in the effects of physical activity on anxiety. Therefore, improving the behavior and awareness of college students’ participation in physical exercise and guiding them to develop regular living habits and correct dietary nutritional patterns can effectively improve and reduce the anxiety level of college students. This kind of regulation provides a valuable reference for the self-management and rehabilitation of college students with anxiety disorders.

## Data availability statement

The raw data supporting the conclusions of this article will be made available by the authors, without undue reservation.

## Ethics statement

The studies involving humans were approved by Faculty of Physical Education, Putian University. The studies were conducted in accordance with the local legislation and institutional requirements. The participants provided their written informed consent to participate in this study. Written informed consent was obtained from the individual(s) for the publication of any potentially identifiable images or data included in this article.

## Author contributions

DS: Writing – review & editing, Writing – original draft, Software, Methodology, Investigation, Formal analysis, Data curation, Conceptualization. XZ: Writing – review & editing, Methodology, Investigation, Data curation, Conceptualization. ZB: Writing – review & editing, Supervision, Investigation, Data curation.
